# Demographic and socioeconomic predictors of religious/spiritual beliefs and behaviours in a prospective cohort study (ALSPAC) in Southwest England: Results from the parental generation

**DOI:** 10.12688/wellcomeopenres.17897.1

**Published:** 2022-05-25

**Authors:** Daniel Major-Smith, Jimmy Morgan, Isaac Halstead, Hamid Reza Tohidinik, Yasmin Iles-Caven, Jean Golding, Kate Northstone

**Affiliations:** 1 MRC Integrative Epidemiology Unit, University of Bristol, Bristol, UK; 2 Centre for Academic Child Health, Population Health Sciences, Bristol Medical School, University of Bristol, Bristol, BS8 2BN, UK; 3 Population Health Sciences, Bristol Medical School, University of Bristol, Bristol, BS8 2BN, UK

**Keywords:** ALSPAC, religion, confounding, bias, socioeconomic position, descriptive study

## Abstract

*Background*: We explored associations between possible demographic and socioeconomic causes of religious/spiritual beliefs and behaviours (RSBB) in the parental generation of the Avon Longitudinal Study of Parents and Children (ALSPAC).

*Methods: *We used a prospective birth cohort study (ALSPAC) in Southwest England with 14,157 enrolled mothers and 14,154 associated partners. Three RSBB outcome measures collected during pregnancy were examined: religious belief (belief in God/a divine power; yes/not sure/no), religious affiliation (Christian/none/other) and religious attendance (frequency of attendance at a place of worship). Multiple demographic and socioeconomic exposures were assessed (23 in mothers and 22 in partners). We explored age-adjusted associations between each exposure and outcome using multinomial regression, in addition to exposure-age interactions.

*Results*: Many demographic and socioeconomic factors were associated with RSBB outcomes, including age, ethnicity, marital status, education, income and deprivation. Overall, higher socioeconomic position was associated with increased levels of RSBB, particularly regarding religious attendance. For instance, compared to mothers with the lowest level of educational attainment, a degree-level education was associated with six-fold increase in the relative risk ratio of religious attendance at least once a week, relative to not attending at all (RRR=5.90; 95% CI=[4.44; 7.86]). The magnitude of these associations often varied by outcome, e.g., income was associated with religious attendance, but not religious affiliation. Although results were demographically and socially patterned, overall effect sizes were relatively small, with a largest pseudo- *R ^2^* value of 2.4%. Patterns of association were similar for both mothers and partners.

*Conclusion*: These findings demonstrate that numerous demographic and socioeconomic factors are associated with RSBB in this population. While additional work is needed to assess whether any of these associations are causal, this descriptive paper can help inform future studies using this data by considering appropriate confounders and thus attempt to minimise bias that confounding may introduce.

## Introduction

There is an increasing appreciation that religious/spiritual beliefs and behaviours (RSBB) may impact health outcomes, both physical and mental ^1– 3^. Despite this growing recognition, the role of RSBB in wider health research is often neglected, partly because of a lack of high-quality prospective studies with detailed information on RSBB and relevant potential confounders ^4^.

Confounding occurs when a third variable causes both the exposure and the outcome ^5– 7^. Factors such as socioeconomic background, education and deprivation – which may cause RSBB – are known to impact health ^8^, and therefore may act as confounders in analyses. Identifying confounders is essential when exploring how RSBB may impact health outcomes as causal inferences may be biased without proper adjustment.

Research has identified three broad categories of variables which may cause RSBB (see 9, 10): socioeconomic, cognition/psychology and cultural transmission. In this paper we focus on the first factor (in addition to demographic factors). A socioeconomic perspective suggests that RSBB may be heightened in times of stress, uncertainty or insecurity as a way for people to explain and understand these events and find meaning in the world ^11– 13^. This perspective posits that as material security increases, such as via well-functioning secular institutions, religiosity will decline. Therefore, socioeconomic factors – such as deprivation, lower social class, lower income and marginalised minority groups – will be associated with heightened religiosity. Some evidence supports this view, finding that as material security increases country-level religiosity declines ^14^, and that marginalised groups in US society, such as women, racial minorities and those from a lower socioeconomic position (SEP), are more likely to be religious ^11^. Indeed, there is evidence that lower SEP, often proxied by educational attainment, is associated with higher levels of religiosity ^15– 17^. However, this effect is not replicated in all studies ^9, 10, 18^, and other research suggests that the association between education and RSBB is more complex (e.g., educational attainment may predict increased church attendance, but reduced levels of prayer), often in denomination-specific ways ^19^.

The aim of this paper is to explore whether demographic and socioeconomic factors are associated with RSBB in the parental generation of a prospective birth cohort (the Avon Longitudinal Study of Parents and Children; ALSPAC) which has detailed data on both RSBB outcomes and sociodemographic variables. This work is therefore primarily descriptive; by examining variables which may cause RSBB we can help inform the choice of confounders in future studies. It is important to note that although there is an underlying assumption throughout this paper that these demographic and socioeconomic factors cause RSBB, it is also possible that these factors are also caused by RSBB, and that causation may be bidirectional. This is a crucial point we return to and expand upon in the discussion. Although previous ALSPAC publications have described this RSBB data and identified broad trends, such as religiosity being higher in both women and the older generation of participants ^20– 23^, to date no study has described these data in detail by exploring various associations between a wide range of demographic and socioeconomic factors and RSBB. Establishing causal links between these variables and RSBB is beyond the scope of this paper, and our aim here is more modest: to describe these broad associations and act as a platform to inform future research in this area.

## Methods

### Participants

Pregnant women resident in Bristol (UK) and surrounding areas with expected dates of delivery between 1 ^st^ April 1991 and 31 ^st^ December 1992 were invited to take part in the study. The initial number of pregnancies enrolled was 14,541, of which there were a total of 14,676 foetuses, resulting in 14,062 live births and 13,988 children who were alive at 1 year of age ^24, 25^. The current research focuses specifically on the parental generation. After removing one pregnancy if the mother had two pregnancies enrolled in ALSPAC (to avoid duplicated data from the same mother) and dropping observations for participants who had withdrawn consent for their data to be used, a total of 14,157 mothers were included in the final dataset, along with 14,154 associated partners (usually the father of the study child; hereafter ‘partners’). Partners were not formally enrolled into ALSPAC, but were given partner-based questionnaires by the mother (if she had a partner and chose to share the questionnaire). This means that partner-based questionnaires may not have been completed by the same partner over time (although numbers of such cases are likely to be relatively small); for the purposes of this study, we assume that the identity of the partner is the same across all waves of data collection used. Although approximately 2,000 partners never participated in ALSPAC, all potential partners have been included here to show levels of missing data, and because many of these partners have information about them from questionnaires completed by the mother. Please note that the study website contains details of all the data that is available through a fully searchable data dictionary and variable search tool.

### Outcome measures

The outcome variables for this study were the participants’ RSBB ( Table 1). These have been measured repeatedly in the parental generation (during pregnancy, at 5, 6, 9 and 28 years post-partum ^22^). For the purposes of this study, we will explore three RSBB outcomes measured at baseline during pregnancy (mean mother’s age at birth = 28.0 [SD = 5.0; range = 15 to 44]; mean partner’s age = 30.4 [SD = 5.8; range = 15 to 70]) which cover a range of religious beliefs and behaviours and have the most complete data: religious belief (belief in God or some divine power; yes vs not sure vs no); religious affiliation (Christian vs none vs other); and religious attendance (frequency of attendance at a place of worship; at least once a week vs at least once a month vs at least once a year vs not at all).

**Table 1.** Summary of religious/spiritual beliefs and behaviours (RSBB) outcomes used in this study for both mothers and partners. Sample sizes are 14,157 for mothers and 14,154 for partners.

RSBB outcome		Mother (N; %)	Partner (N; %)
**Belief in God/a divine power**	*Yes*	6,067 (49.9%)	3,552 (36.9%)
	*Not sure*	4,289 (35.3%)	3,311 (34.4%)
	*No*	1,806 (14.8%)	2,758 (28.7%)
	*Total*	12,162	9,621
Missing data		1,995 (14.1%)	4,533 (32.0%)
**Religious affiliation**	*None*	1,836 (15.3%)	2,440 (25.8%)
	*Christian*	9,666 (80.5%)	6,521 (68.9%)
	*Other*	511 (4.2%)	506 (5.3%)
	*Total*	12,013	9,467
Missing data		2,144 (15.1%)	4,687 (33.1%)
**Frequency of attendance at a church/place** **of worship**	*Min once a week*	877 (7.4%)	570 (6.1%)
	*Min once a month*	817 (6.9%)	406 (4.3%)
	*Min once a year*	3,480 (29.3%)	2,473 (26.2%)
	*Not at all*	6,715 (56.5%)	5,974 (63.4%)
	*Total*	11,889	9,423
Missing data		2,268 (16.0%)	4,731 (33.4%)

### Exposure measures

To explore the demographic and socioeconomic factors associated with RSBB we used a range of exposures chosen according to empirically or theoretically supported relationships or *a priori* reasoning of potential causal relationships with RSBB. This encompasses socioeconomic factors described in the introduction, in addition to a broad demographics category. A summary of these variables is given in Table 2, while full descriptive statistics of each exposure are provided in Table S1 (please see Extended data for supplementary tables and figures ^26^). All exposures were assessed during pregnancy or shortly afterwards.

**Table 2.** Summary of variables used as exposures. Other than household income (measured when study children were aged 3/4 years) and partner financial difficulties (measured 8 months post-partum), all variables were assessed in pregnancy or shortly after.

Variable ( *variable name*)	Variable coding	Notes
*Demographic variables*		
Age ( *AgeAtBirth*; *AgeInPreg* for partners)	Continuous (years)	
Ethnicity ( *nonWhiteEthnic*)	Binary (White vs Other than White)	Also used recent COVID4 questionnaire to fill in missing data
Marital status ( *maritalStatus*)	Unordered category (never married vs currently married vs widowed/divorced/separated)	
Residential mobility (in last 5 years; *mobility*)	Ordered category (0 moves vs 1 move vs 2 moves vs 3 moves vs 4 moves vs 5 or more moves)	
Urban/rural status ( *rural*)	Binary (town/village/hamlet vs urban)	For partners, using mothers data
Parity ( *parity*)	Ordered category (0 vs 1 vs 2 or more)	For partners, using mothers data
*Socioeconomic variables*		
Highest education qualification ( *education*)	Ordered category (CSE/none vs vocational vs O-level vs A-level vs degree) ^a^	
Mother’s highest education qualification ( *maternalEdu*)	Ordered category (CSE/none vs vocational vs O-level vs A-level vs degree) ^a^	
Father’s highest education qualification ( *paternalEdu*)	Ordered category (CSE/none vs vocational vs O-level vs A-level vs degree) ^a^	
Occupational social class ( *highSocClass*)	Binary (low [III manual/IV/V] vs high [I/II/III non-manual]) ^b^	
Mother’s occupational social class ( *highSocClass_mat*)	Binary (low [III manual/IV/V] vs high [I/II/III non-manual]) ^b^	
Father’s occupational social class ( *highSocClass_pat*)	Binary (low [III manual/IV/V] vs high [I/II/III non-manual]) ^b^	
Household income ( *income*)	Continuous (log income per/week)	
Index of multiple deprivation ( *IMD*)	Ordered category (1 ^st^ quintile [least deprived] vs 2 ^nd^ quintile vs 3 ^rd^ quintile vs 4 ^th^ quintile vs 5 ^th^ quintile [most deprived])	For partners, using mothers data
Townsend deprivation index ( *townsendDep*)	Ordered category (1 ^st^ quintile [least deprived] vs 2 ^nd^ quintile vs 3 ^rd^ quintile vs 4 ^th^ quintile vs 5 ^th^ quintile [most deprived])	For partners, using mothers data
Mother or partner access to car ( *accessToCar*)	Binary (yes vs no)	
Housing status ( *housing*)	Unordered category (owned/mortgaged vs renting vs council/housing association vs other)	For partners, using mothers data
Recent financial difficulties ( *financeDiffs*)	Binary (yes vs no)	
Financial difficulties score ( *financeDiffsScore*)	Continuous (from 0 [no difficulties] to 15 [severe difficulties])	
Family’s financial circumstances got worse during childhood ( *poorerChildhood*)	Binary (yes vs no)	
Crowding index ( *crowding*)	Ordered category (calculated by dividing the number of people in the household by the number of rooms; ≤ 0.5; > 0.5 to 0.75; > 0.75 to 1; > 1)	For partners, using mothers data
Self-reported neighbourhood quality index ( *neighPercept*)	Continuous (score from 0 [low quality neighbourhood] to 12 [high quality neighbourhood])	
Partner absence in pregnancy ( *partnerAbsence*)	Binary (partner present vs partner absent)	Not applicable for partners

^a^ CSE = Certificate of Secondary Education qualification (examinations sat at the end of secondary school at approx. age 16; compulsory from the early 1970s, unless completing O-level qualifications instead); O-level = Ordinary level qualifications (examinations sat at the end of secondary school, often for more academically-able pupils at approx. age 16); A-level = Advanced level qualification (non-compulsory examinations sat at the end of college or sixth form at approx. age 18).

^b^ For more information on these occupational social classes, see: https://sru.soc.surrey.ac.uk/SRU9.html.

### Confounder variables

As the aim of this paper is to describe broad associations of factors which may cause RSBB, rather than provide a causal estimate of these relationships, all analyses here only adjust for age (other than the age-only models). Further research is required to explore these relationships in more depth to make causal claims, but adjusting for age will remove one common source of confounding.

### Analysis

We first explored correlations between the exposures to examine how inter-related these variables were. For all continuous, ordered categorical and binary variables we used Pearson correlations, while for unordered categorical variables (only two variables; home ownership and marital status) we approximated these correlation coefficients by running a series of multinomial models with these variables as the outcome and then square-rooting the pseudo- *R ^2^* value (cf. 27). While ordinal and binary variables do not meet the assumptions for Pearson correlations (i.e., they are not continuous or normally-distributed), as this approach was used primarily to understand the broad associations between these variables – rather than the specific correlation coefficients – we believe it is appropriate for our purposes here.

We then assessed whether each of the exposures in Table 2 was associated with each of the RSBB outcomes in Table 1 using multinomial regression. Multinomial analyses were chosen because two of the outcomes (religious belief and religious affiliation) were unordered categorical variables. We also decided to run multinomial regression on the ordered categorical outcome (religious attendance) for two reasons. First, we initially ran ordinal regression models on this outcome, but the assumption of proportional odds was violated (as indicated via a Brant test); multinomial regression does not require this assumption. Second, by performing multinomial regressions on all outcomes the regression coefficients are all on the same scale (relative risk ratios) and therefore broadly comparable to one another, facilitating interpretation of effect sizes. All analyses adjusted for age (other than the age-only models). Given differences in RSBB by age/generation ^20^, we also explored whether predictors of RSBB varied by age by including an interaction between age and each exposure (assuming a linear association with age).

To provide a single *p*-value for each model to assess model fit, we ran two sets of likelihood ratio tests for each exposure-outcome combination: the first assessed whether inclusion of the exposure improved model fit relative to an age-only model (or an empty model, where age was the exposure); the second assessed whether inclusion of an interaction term between age and the exposure improved model fit relative to the model with no interaction. In an attempt to minimise the false discovery rate, for each outcome we applied a Bonferroni-correction corresponding to the number of exposures tested. For mothers there were 23 exposures, giving a Bonferroni-adjusted threshold when using a standard 0.05 alpha value of 0.05/23 = 0.0022 (0.05/22 = 0.0023 for the interaction models); as partners had 22 exposures, the adjusted alpha value was 0.05/22 = 0.0023 (0.05/21 = 0.0024 for the interaction models). These adjusted thresholds were not used to arbitrarily dichotomise results into ‘significant’ and ‘non-significant’ ^28^, but rather were used as a useful summary to describe large numbers of associations and to assess the strength of evidence against the null hypothesis of no association between the exposure and outcome ^29^. To give an indication of the increase in model fit resulting from inclusion of the exposure, we calculated the difference in McFadden’s pseudo- *R ^2^* value between the model with vs without the exposure (or with vs without the interaction term, for interaction models). Although this pseudo- *R ^2^* value is not directly comparable to a standard *R ^2^* ‘variance explained’ statistic from a linear model (pseudo- *R ^2^* values often being smaller than the corresponding *R ^2^* value, for example 30, 31), it is nonetheless a useful metric to assess model fit and for comparisons between exposures. This approach was repeated in the mother and partner cohorts. All analyses were conducted in Stata v.17, but can also be performed in the open-source software R ^32^. 

## Results

### Descriptive statistics

Descriptive statistics for the RSBB outcomes are displayed in Table 1. In the mother’s cohort, 50% believed in God/a divine power, while 15% did not; religious belief was lower among partners (37%), while non-belief was higher (29%). Patterns were similar for religious affiliation, with 80% of mothers having a Christian affiliation and 15% having no affiliation; for partners, 69% identified as Christian and 26% had no affiliation. Religious attendance was lower than religious belief and affiliation, with 56% of mothers and 63% of partners never attending a place of worship; 14% of mothers and 10% of partners attended a place of worship a minimum of once a week or once a month. Descriptive statistics for each of the exposures, split by each RSBB outcome category, are displayed in Tables S2 (for religious belief), S3 (for religious affiliation) and S4 (for religious attendance).

### Mothers

**Figure 1.**
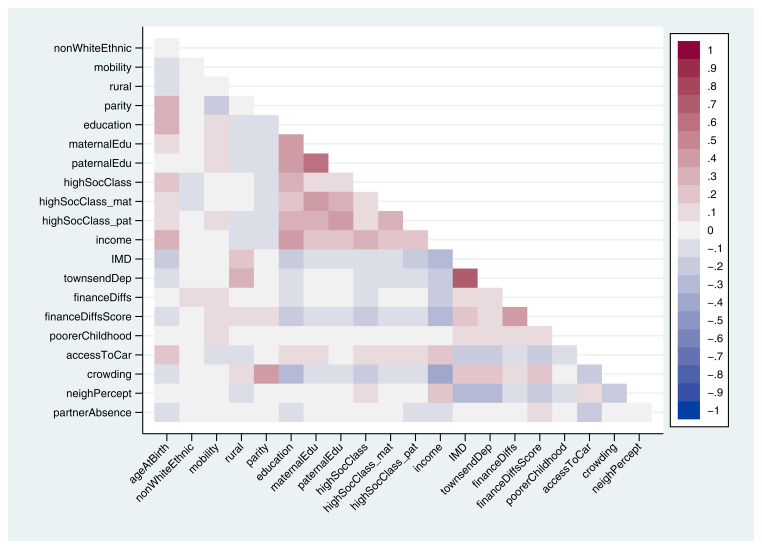
Heat-plot of the correlation matrix between all continuous, ordered categorical and binary exposures used in the mother’s analysis (numerical results are displayed in table S5). For full details on the variables included here, see Table 2.

A heat-plot of the correlation matrix between all 21 continuous, ordered categorical and binary variables is displayed in Figure 1 (full correlation coefficients are displayed in Table S5; approximate correlation coefficients for the unordered categorical variables home ownership status and marital status are in Table S6). Other than a few highly-correlated variables measuring similar constructs – such as IMD (index of multiple deprivation) and Townsend deprivation indices – and clusters based on education and occupational social class, associations between most of the exposures were not especially strong. For instance, the correlation between the mother’s highest educational qualification and the highest qualification of her mother was 0.41, while income was negatively associated with index of multiple deprivation ( *r* = -0.32; although given the issues raised above regarding using Pearson correlations for binary and ordinal variables, these specific coefficients should be taken as merely illustrative of the strength of these relationships). This suggests that, although many of the exposures are correlated to some extent, overall, they are likely to be at least somewhat independent.

**Figure 2.**
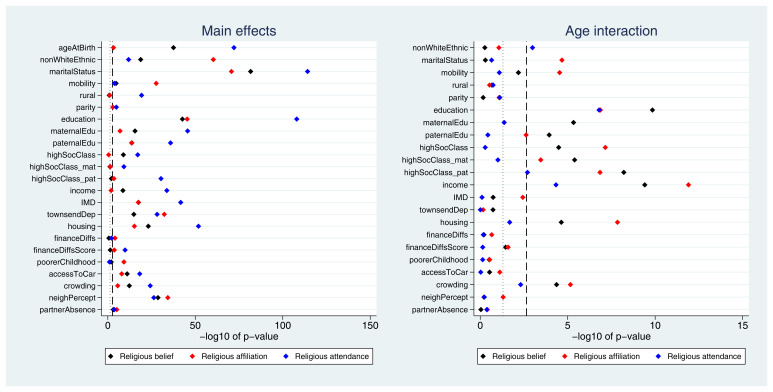
*P*-values for each exposure and RSBB outcomes for mothers. The left-hand plot shows the age-adjusted main effects; the right-hand plot shows the interaction between age and the exposure. The light dashed line indicates a standard 0.05 *p*-value threshold; the thicker dashed line denotes the Bonferroni-corrected *p*-value threshold (0.05/23 = 0.0022 for main effects and 0.05/22 = 0.0023 for interaction effects). Results to the right of these lines indicate a *p*-value below said threshold. For full details on the variables included here, see Table 2. For sample sizes, see tables S9-S11.

Many exposures were associated with each of the three RSBB outcomes. A plot of the *p*-values from the likelihood ratio tests is displayed in Figure 2. Taking ‘religious belief’ as an example, 17 of 23 (74%) exposure main effects were associated with this outcome at the Bonferroni-corrected alpha value, while 20 (87%) reached a conventional 0.05 alpha threshold. Compared to main effects, there were fewer associations reported for interaction terms; again using ‘religious belief’ as an example, 9 of 22 (41%) interactions were associated at the Bonferroni-corrected alpha value, while 11 (50%) reached a 0.05 alpha threshold. Results were broadly comparable for the religious affiliation outcome, although for religious attendance more main effects were reported (but fewer interaction associations). A summary of results for each of the RSBB outcomes is in Table 3 (with a full list of *p*-values from all likelihood ratio tests given in Table S7).

**Table 3.** Summary of associations between 23 exposures and the three RSBB outcomes at both the Bonferroni-corrected and conventional 0.05 alpha levels for the mothers’ analyses. Results show the number (percentage) of exposures below both the Bonferroni-corrected and 0.05 alpha levels for each RSBB outcome.

	Number (%) of main effects below *p*-value thresholds		Number (%) of interactions below *p*-value thresholds	
	Bonferroni-corrected (0.05/23 = 0.0022)	0.05	Bonferroni-corrected (0.05/22 = 0.0023)	0.05
Religious belief	17 (74%)	20 (87%)	9 (41%)	11 (50%)
Religious affiliation	19 (83%)	21 (91%)	9 (41%)	14 (62%)
Religious attendance	21 (91%)	22 (96%)	4 (18%)	7 (32%)

**Figure 3.**
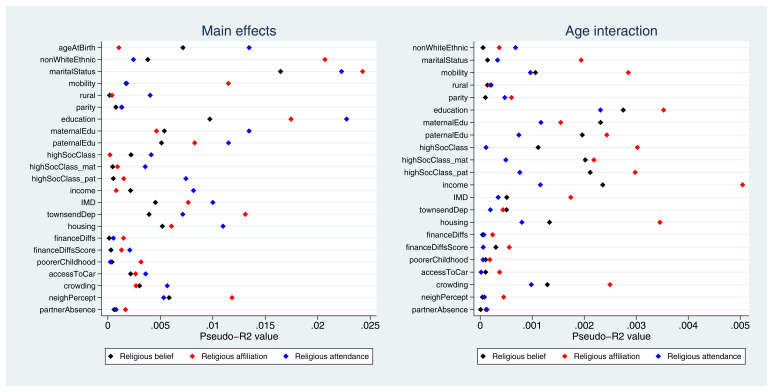
Pseudo- *R ^2^* values for each exposure and RSBB outcomes for mothers. The left-hand plot shows the age-adjusted main effects; the right-hand plot shows the interaction between age and the exposure. For full details on the variables included here, see Table 2. For sample sizes, see tables S9-S11.

Pseudo- *R ^2^* values for each exposure-outcome association are displayed in Figure 3. While pseudo- *R ^2^* values cannot be interpreted directly as measures of variance explained, overall these results demonstrate that the improvement in model fit due to each exposure is relatively small. The highest pseudo- *R ^2^* value is 2.4% (with marital status as the exposure and religious affiliation as the outcome), and the majority of values are below 1%. Pseudo- *R ^2^* values for the interaction terms are even weaker, with the largest value of 0.5% for the interaction between age and income with religious affiliation as the outcome. Thus, although the majority of exposures were below the Bonferroni-adjusted *p*-value threshold, the amount of variance explained by these exposures is likely to be small (full pseudo- *R ^2^* results are given in Table S8). Overall, these findings suggest that many exposures were associated with these RSBB outcomes, albeit relatively weakly, and that fewer interaction effects with age were reported.

However, this focus on *p*-values and pseudo- *R ^2^* values only tells us whether an association is present and the strength of the association, and not the direction of said association. Additionally, as can be seen in Figure 2 and Figure 3, there appears to be some heterogeneity of effects between different RSBB outcomes; for instance, age at birth is strongly associated with religious belief and religious attendance, but less so for religious affiliation. We now turn to specific parameter estimates to explore the direction of these results. Given the sheer number of associations explored here, we will pick out a few key results to focus on (full results are given in Tables S9-S11).

**Figure 4.**
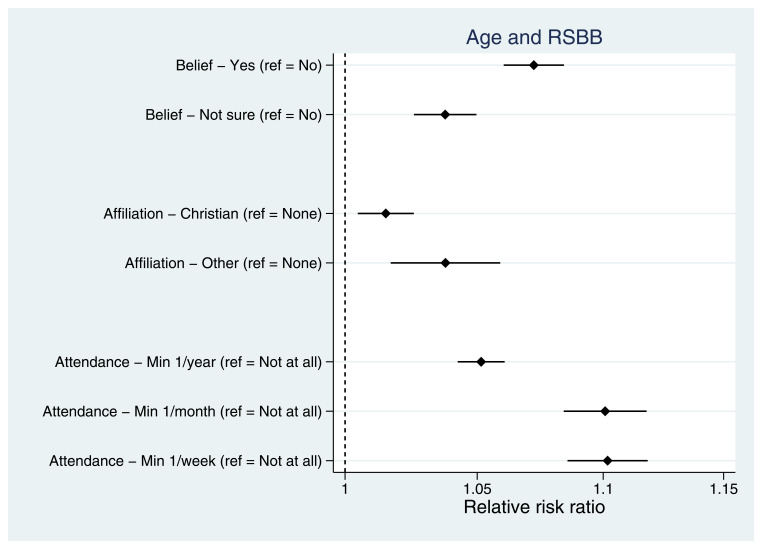
Associations between age and RSBB outcomes for mothers. All results are from multinomial regression analyses and show the relative risk ratio for a one-year increase in age relative to the outcome reference (specified on the y-axis). The x-axis is on the log scale. Error bars are 95% confidence intervals. Sample sizes: religious belief = 12,106; religious affiliation = 11,959; religious attendance = 11,836.

Taking demographic variables first, older mothers were more religious than younger mothers, with older mothers more likely to believe in God/a divine power, have a religious affiliation and attend a place of worship more frequently ( Figure 4). As relative risk ratios from multinomial regressions are not necessarily intuitive to interpret, predicted probabilities for each of the RSBB outcomes by age are shown in Figure S1. Having an ethnicity other than White (Figure S2), being married (relative to never being married; Figure S3) and lower levels of residential mobility (Figure S4) were each associated with increased religiosity. Urban/rural status and parity had little association with RSBB.

**Figure 5.**
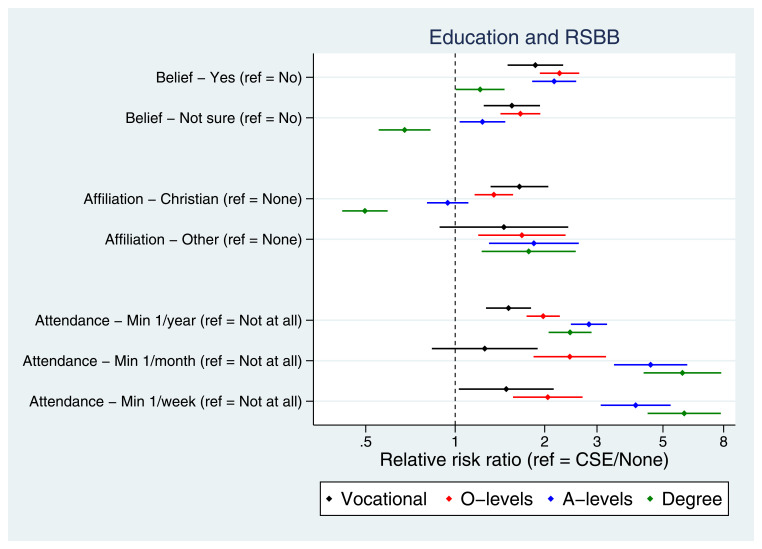
Associations between education and RSBB outcomes for mothers. All results are from multinomial regression analyses and show the relative risk ratio for a given educational level relative to both the educational reference level (CSE/None) and the outcome reference (specified on the y-axis). The x-axis is on the log scale. Error bars are 95% confidence intervals. Sample sizes: religious belief = 11,456; religious affiliation = 11,326; religious attendance = 11,206.

Many socioeconomic factors were associated with RSBB. For instance, education was strongly associated with RSBB, albeit sometimes in a non-linear fashion ( Figure 5); higher educational attainment was associated with an increased probability of both religious belief and Christian religious affiliation, although this reversed for mothers with a degree. In contrast, religious attendance had a broadly linear association with education, with higher educational qualifications associated with increased attendance. Living in owned/mortgaged accommodation (Figure S5), lower levels of deprivation (Figure S6), higher income (Figure S7) and higher occupational social class (Figure S8) were each associated with higher levels of religiosity. Factors such as parental social class, recent financial difficulties, partner absence during pregnancy or family becoming poorer in childhood had weaker and/or inconsistent associations with RSBB.

Overall, there were few interactions between age and RSBB. Exceptions include education, where older mothers with a degree were less likely to believe in God/a divine power, identify as Christian or attend a place of worship, compared to younger mothers with a degree (Figure S9 for relative risk ratios; figures S10a, S10b and S10c for predicted probabilities by age and education for these outcomes). Additionally, interactions between age and both income and occupational social class were found, with higher income and occupational social class associated with being less likely to believe in God or identify as Christian among older mothers (Figures S11 and S12).

### Partners

A heat-plot of the correlation matrix between the 20 continuous, ordered categorical and binary variables for partners is displayed in Figure S13 (full correlation matrix in Table S12; approximate correlation coefficients for the unordered categorical variables home ownership status and marital status are in Table S13). As with the mothers’ data, other than some clustering by the deprivation and education/occupational social class variables, the majority of associations between the exposures were moderate or weak (e.g., the correlation between the partner’s highest educational qualification and being of higher occupational social class was 0.41).

**Figure 6.**
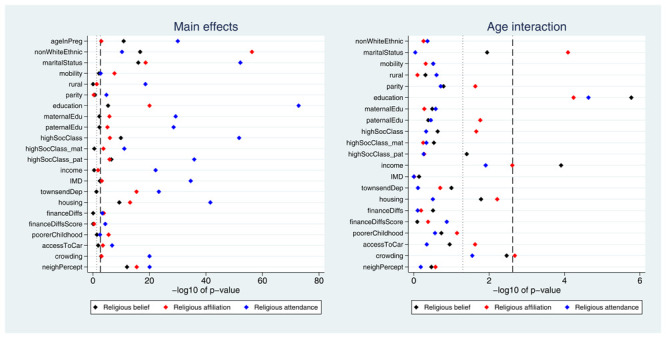
*P*-values for each exposure and RSBB outcomes for partners. The left-hand plot shows the age-adjusted main effects; the right-hand plot shows the interaction between age and the exposure. The light dashed line indicates a standard 0.05 *p*-value threshold; the thicker dashed line denotes the Bonferroni-corrected *p*-value threshold (0.05/22 = 0.0023 for main effects and 0.05/21 = 0.0024 for interaction effects). Results to the right of these lines indicate a *p*-value below said threshold. For full details on the variables included here, see Table 2. For sample sizes, see tables S16-S18.

Many exposures were associated with each of the RSBB outcomes. A plot of the *p*-values from the likelihood ratio tests is displayed in Figure 6. A summary of results for each of the RSBB outcomes is in Table 4, with a full list of *p*-values from all likelihood ratio tests in Table S14. Similar to the mothers’ data, many main effects were identified, few interaction effects were reported, and there was heterogeneity in terms of exposure associations over different RSBB outcomes. The pseudo- *R ^2^* values are displayed in Figure S14 (full results in Table S15), and are again relatively small in magnitude and similar to the mothers’ data (largest main effect pseudo- *R ^2^* of 2.4%; largest interaction pseudo- *R ^2^* of 0.3%).

**Table 4.** Summary of associations between 22 exposures and the three RSBB outcomes at both the Bonferroni-corrected and conventional 0.05 alpha levels for the partners analyses. Results show the number (percentage) of exposures below both the Bonferroni-corrected and 0.05 alpha levels for each RSBB outcome.

	Number (%) of main effects below *p*-value thresholds		Number (%) of interactions below *p*-value thresholds	
	Bonferroni-corrected (0.05/22 = 0.0023)	0.05	Bonferroni-corrected (0.05/21 = 0.0024)	0.05
Religious belief	9 (41%)	15 (68%)	2 (10%)	6 (29%)
Religious affiliation	18 (82%)	20 (91%)	3 (14%)	9 (43%)
Religious attendance	21 (95%)	22 (100%)	1 (5%)	3 (14%)

We now turn to the direction of these partner results. Again, given the large number of associations explored, we will only focus on a few key results here (full results are given in Tables S16-S18). Similar to the mothers’ data, of the demographic variables, older age (Figure S15 for relative risk ratios; Figure S16 for predicted probabilities), other than White ethnicity (Figure S17), being married (Figure S18) and lower levels of residential mobility (Figure S19) were associated with increased religiosity among the partners. For socioeconomic factors, education was again associated with RSBB, with higher educational qualifications associated with being less likely to identify as Christian, yet increased religious attendance (Figure S20). Higher occupational social class (figure S21), lower levels of deprivation (Figure S22) and home ownership (Figure S23) were associated with increased religiosity. Other socioeconomic factors had null or inconsistent associations with RSBB, such as higher income being associated with increased religious attendance but not religious belief or affiliation (Figure S24).

As with the mothers, there were few interactions between age and the exposures, although older partners with a degree were less likely to believe in God/divine power, identify as Christian or attend a place of worship, compared to younger partners with a degree (Figure S25 for relative risk ratios; Figures S26a, S26b and S26c for predicted probabilities by age and education for these outcomes). Higher income was also associated with being less likely to believe in God among older partners (Figure S27).

## Discussion

Many demographic and socioeconomic factors are associated with RSBB in mothers and partners in this cohort. Older age, other than White ethnicity, being married, higher educational attainment, increased income, higher occupational social class and lower deprivation were all associated with increased religiosity, for at least some RSBB outcomes. Other factors, such as parity, recent financial difficulties, rural vs urban location, partner absence (for mothers), access to a car and family becoming poorer during the parent’s childhood had either weak, null or inconsistent associations with RSBB outcomes. Despite some highly significant associations (as indicated by *p*-values), the estimated variance explained using pseudo- *R ^2^* values was relatively low, with a maximum value of 2.4%, highlighting that relatively little of the variation in RSBB is explained by individual demographic and socioeconomic factors. Patterns of results were broadly similar for both mothers and partners, although religiosity was lower among partners ( Table 1), as reported previously ^23^.

Given the descriptive nature of this work we caution against interpreting these results in causal terms; however, we make some tentative comparisons with previous research to situate these results in the wider literature. First, contrary to many previous studies ^11, 15, 16^, we found a positive association between many socioeconomic factors and RSBB outcomes, with increased household income, lower levels of deprivation and higher occupational social class associated with increased religious belief, affiliation and attendance. These patterns were similar, but more nuanced, for education, with higher educational attainment linearly associated with religious attendance, but non-linearly associated with religious belief and affiliation; relative to the lowest education category (CSE/no qualifications), mothers with vocational, O-level or A-level qualifications were more likely to believe in God or have a religious affiliation, while mothers with a degree were less likely to have religious belief or state they had a Christian religious affiliation ( Figure 5; similar patterns were observed in the partners, Figure S20). Together, these results suggest that lower socioeconomic position is not associated with increased religiosity in this population. Additional research is required to understand these socially patterned results in greater detail and why they differ from previous research. Potential explanations include society-level differences in religiosity and its association with education ^16^, with much previous work conducted in North America ^11, 15^, and differences in how RSBB and education are defined and operationalised (e.g., religious belief vs affiliation vs attendance, or highest educational qualification [categorical] vs university degree [binary] vs years of education [continuous]).

Other socioeconomic factors – such as recent financial difficulties, access to a car, partner absence (for mothers) and family financial difficulties in childhood – had weaker associations with RSBB outcomes than wider socioeconomic factors such as education, deprivation and occupational social class. These broader socioeconomic factors may therefore have a larger impact on religious beliefs and behaviours in this population (assuming that these socioeconomic factors cause RSBB).

We also observed variation in the associations between exposures and different RSBB outcomes. For instance, among mothers, both age ( Figure 4) and income (Figure S7) were associated with religious belief and religious attendance, but associations with religious affiliation were weaker. These results indicate that there may be heterogeneity across RSBB outcomes and exposures, suggesting that different RSBB variables measure different facets of religiosity, each of which may be caused by different factors. For instance, religious belief and attendance may be somewhat independent of religious affiliation ^33^, as affiliation may reflect nominal or historical group identity, rather than strength of religious convictions. However, when comparing these results we need to be aware that the difference between ‘significant’ and ‘non-significant’ may not itself be significant ^34^. That is, just because higher occupational social class (as an example) is ‘significantly’ associated with religious belief, but ‘non-significantly’ associated with religious affiliation, this does not mean that the difference between occupational social class for religious belief and religious affiliation are ‘significantly’ different from one another (in fact, the effect sizes are broadly similar; Figure S8). Similar considerations also apply when comparing different exposures within one RSBB outcome as well. Nonetheless, despite these caveats there does appear to be some variation between different RSBB outcomes that may help inform future work and could be explored in greater detail.

Although less prevalent than main effects, some interaction effects with age were reported. The strongest effects were found for education and income. Older participants with higher educational attainment and income were associated with lower levels of religiosity compared to younger participants. These results demonstrate that associations between the exposure and RSBB outcome may vary by the age of the mother or partner, so should be considered when using this data (although the variance explained by these interaction terms is rather weak; maximum pseudo- *R ^2^* of 0.5%).

### Strengths and limitations

A key strength of this research is the use of a large, deeply-phenotyped, longitudinal birth cohort with a wealth of variables measured which could be used as confounders in future studies. This cohort also contains detailed, longitudinal RSBB data, which can be used to explore associations between RSBB and health outcomes in detail. At recruitment in pregnancy, this cohort was broadly representative of the target population ^24^, although since recruitment there has been drop-out, which is known to be non-random and may result in selection bias ^35– 37^.

There are several limitations with this study. First, we attempted to focus on demographic and socioeconomic exposures that may *plausibly* cause RSBB. In many cases, however, it is not certain whether the exposure variable is a cause or consequence of RSBB, or both (or neither). For instance, marital status may cause RSBB, but it is also possible that being religious causes people to be more likely to get and remain married; thus, there may be reciprocal causation, with religiosity causing an increased probability of getting/staying married, and then marriage increasing subsequent religiosity. As another example, certain religions may encourage (or discourage) norms and behaviours which promote educational attainment, again meaning that RSBB would be a cause, rather than consequence, of socioeconomic factors. These issues may also play out on longer historical timescales as well; for instance, due to religious discrimination and persecution, religious affiliation may result in differences in socioeconomic position. While some factors cannot be caused by RSBB, such as age, sex or ethnicity, these potential issues of reverse and bidirectional causality may apply to many of the variables explored here. One consequence of this is that unravelling the factors causing RSBB becomes a difficult task; if a variable is caused by RSBB, then we would not want to include it in a model aiming to examine the causes of RSBB, as doing so may also have the unintended side effect of acting as a collider ^5, 38, 39^, thus biasing other causal estimates; see Figure S28 for a simple worked illustration.

A second consequence of this potential reciprocal causation is that this has implications for our choice of variables when trying to estimate causal effects if RSBB is the exposure. For example, say that we are interested in whether RSBB impacts mental health, and we are deciding whether to include marital status as a confounder or not ^5, 7, 40^. If marital status causes both RSBB and mental health, then it is a confounder and should be adjusted for to obtain an unbiased effect of RSBB on mental health (in this example we are ignoring all other potential confounders; Figure S29a). If RSBB causes both marital status and mental health, then marital status may be a mediator on the RSBB-mental health causal path, and we would not want to adjust for this if we were interested in the total causal effect of RSBB on mental health (Figure S29b). If there is reciprocal causation, with RSBB at time 1 causing an increased probability of marriage, which in turn increases RSBB at time 2, which in turn increases the probability of staying married, then we would have a situation where marital status is both a confounder (at time 1) and a mediator (at time 2; Figure S29c). If marital status was not measured at time 1, then estimating a causal effect of RSBB on mental health may be impossible using standard multivariable regression-based approaches. The causal model generating the observed data therefore needs to be considered, and the analysts’ assumptions made clear, when deciding which covariates to include in an analysis model ^5^. This paper has focused on RSBB data from one time-point in each analysis; where possible, future work can help untangle these thorny issues by making use of the longitudinal and intergenerational nature of the ALSPAC data with repeated data on RSBB and many of the exposures here in both the parental and child generations (for a discussion of similar causal considerations when working with longitudinal data, see 41).

A further limitation is that as this paper is descriptive and only adjusted for age, it is possible that many associations may be biased due to residual confounding. For example, both age and education are likely to cause both income and RSBB, yet as we did not adjust for education here when exploring income, this association may be biased. However, as we have repeatedly stressed, the aim of this paper is purely to describe these patterns and inform future work, and these associations should not be taken as causal estimates. Missing data may also result in bias due to selection. This could occur if both the exposure and the outcome, or unmeasured factors associated with both, cause selection/participation ^5, 42^; we are currently exploring whether RSBB is associated with continued ALSPAC participation (and hence selection). Methods such as multiple imputation ^43^, inverse-probability weighting ^44^, and sensitivity analyses ^45^ could be used to explore/test these assumptions. Resolving these issues is beyond the scope of this paper, but concerns of bias due to confounding and selection need to be explored in future studies using this data, especially when the aim is causal inference.

A further limitation is that it is difficult to know how generalisable these results are. Despite differences in religiosity, associations between exposures and RSBB outcomes for mothers and partners in this study were broadly similar, but whether the same factors would be associated with RSBB in different generations (e.g., their children; analysis of which is currently underway), historically, or across nations, religions and cultures is difficult to say. However, this cultural, social and historical variation is likely to be substantial ^16, 46^. Therefore, analyses similar to these need to be replicated in independent cohorts both in the UK and cross-culturally before making broad generalisations about the factors associated with RSBB, and to understand the social, cultural and ecological factors shaping these relationships.

Finally, we note that this paper does not cover all potential causes of RSBB. For instance, as mentioned in the introduction, studies have suggested three broad categories of factors which may cause RSBB: socioeconomic, cognitive/psychological and cultural transmission (in addition to demographic ^9, 10^). Here we have only focused on demographic and socioeconomic variables; additional descriptive work in this cohort is currently underway exploring cognitive/psychological and cultural factor associated with RSBB, while other research is examining the potential influence of environmental exposures, such as cigarette smoking and traumatic events, on RSBB.

## Conclusion

These results demonstrate that numerous demographic and socioeconomic factors are associated with religious/spiritual beliefs and behaviours among this cohort of Bristol-based mothers and partners, particularly: age, ethnicity, marital status, education, income, occupational social class and deprivation. In general, higher socioeconomic position is associated with greater religiosity. However, individually these variables explain relatively little of the variation in RSBB. We again emphasise that these results should not be interpreted causally; nonetheless, we hope that this descriptive paper can be used to help inform future studies using this data, particularly regarding differences between the RSBB outcomes and the choice of potential demographic and socioeconomic confounders.

## Ethics

Ethical approval for the study was obtained from the ALSPAC Ethics and Law Committee and the Local Research Ethics Committees. Informed consent for the use of data collected via questionnaires and clinics was obtained from participants following the recommendations of the ALSPAC Ethics and Law Committee at the time.

## Data availability

### Underlying data

Please see the ALSPAC data management plan which describes the policy regarding data sharing ( http://www.bristol.ac.uk/alspac/researchers/data-access/documents/alspac-data-management-plan.pdf), which is by a system of managed open access. Data used for this submission will be made available on request to the Executive ( alspac-exec@bristol.ac.uk). The datasets presented in this article are linked to ALSPAC project number B3911, please quote this project number during your application. Analysis code supporting this submission is openly-available at: https://github.com/djsmith-90/AnalysisCode_PredictorsOfRSBB_B3911.

The steps below highlight how to apply for access to the data included in this study and all other ALSPAC data:

   1. Please read the ALSPAC access policy ( http://www.bristol.ac.uk/media-library/sites/alspac/documents/researchers/data-access/ALSPAC_Access_Policy.pdf) which describes the process of accessing the data and samples in detail, and outlines the costs associated with doing so.

   2. You may also find it useful to browse our fully searchable research proposals database ( https://proposals.epi.bristol.ac.uk/?q=proposalSummaries), which lists all research projects that have been approved since April 2011.

   3. Please submit your research proposal ( https://proposals.epi.bristol.ac.uk/) for consideration by the ALSPAC Executive Committee. You will receive a response within 10 working days to advise you whether your proposal has been approved.

### Extended data

Open Science Framework: Supplementary information supporting this submission can be found on the Open Science Framework “Demographic and socioeconomic predictors of religious/spiritual beliefs and behaviours in a prospective cohort study (ALSPAC) in Southwest England: Results from the parental generation” project page, https://www.doi.org/10.17605/OSF.IO/NYJWH ^26^.

This project contains the following extended data:

- “G0SocioDemoPredictorsOfRSBB_SuppInfo.pdf” (the supplementary information file)
- “G0SocioDemoPredictorsOfRSBB_STROBE.pdf” (the completed STROBE cohort study reporting guidelines checklist).

Data are available under the terms of the Creative Commons Attribution 4.0 International license (CC-BY 4.0).
