# Supplementary material for: Demographic and socioeconomic predictors of religious/spiritual beliefs and behaviours in a prospective cohort study (ALSPAC) in Southwest England: Results from the parental generation
Source: Wellcome Open Res. 2023 Aug 22;7:159. Originally published 2022 May 25. [Version 3] doi: 10.12688/wellcomeopenres.17897.3 (PMC10410183; doi:10.12688/wellcomeopenres.17897.3)
Supplement: Peer review history

# Reviewer report: Reviewer response for version 3

**DOI:** 10.21956/wellcomeopenres.22081.r65743

**Published:** 2023-09-19

**Authors:** Leor Zmigrod

**Affiliations:** 1 Department of Psychology, University of Cambridge, Cambridge, UK

Confirming the revisions are satisfactory.

Is the work clearly and accurately presented and does it cite the current literature?

Yes

If applicable, is the statistical analysis and its interpretation appropriate?

Yes

Are all the source data underlying the results available to ensure full reproducibility?

Partly

Is the study design appropriate and is the work technically sound?

Yes

Are the conclusions drawn adequately supported by the results?

Yes

Are sufficient details of methods and analysis provided to allow replication by others?

Yes

Reviewer Expertise:

NA

I confirm that I have read this submission and believe that I have an appropriate level of expertise to confirm that it is of an acceptable scientific standard.

# Reviewer report: Reviewer response for version 2

**DOI:** 10.21956/wellcomeopenres.20786.r62264

**Published:** 2023-08-07

**Authors:** Rana Jawad

**Affiliations:** 1 Department of Social & Policy Sciences, University of Bath, Bath, England, UK

The paper explores associations between possible demographic and socioeconomic causes of religious/spiritual beliefs and behaviours (RSBB) in the parental generation of the Avon Longitudinal Study of Parents and Children (ALSPAC). It uses a prospective birth cohort study (ALSPAC) in Southwest England with 14,157 enrolled mothers and 14,154 associated partners. **Three RSBB outcome measures collected during pregnancy are examined: religious belief (belief in God/a divine power), religious affiliation (Christian/none/other) and religious attendance. Multiple demographic and socioeconomic exposures are. exploring age-adjusted associations between exposures and outcomes using multinomial regression, in addition to exposure-age interactions.

 The study finds that a range of demographic and socioeconomic factors are associated with RSBB, including age, ethnicity, marital status, education, income and deprivation. A surprising and novel finding is that  higher socioeconomic position (including degree-level education) is associated with increased levels of RSBB, particularly regarding religious attendance. The depth of these associations vary by outcome, for example, income is strongly associated with religious attendance, but only weakly with religious affiliation. Although results were demographically and socially patterned, overall effect sizes are small. What is note-worthy about the results is that the observed positive association between socioeconomic position and RSBB goes counter to much theoretical and empirical work. Reasons given by the authors for these differences are discussed, including cross-cultural variation (mainly between the UK and USA) in religiosity and state support, and differences between RSBB measures. One reason not explored in the paper is the close social and political association between the Church of England and the British middle class which might account for the findings regarding higher socio-economic status. This does not however, help to explain ethnic minority religious beliefs and orientations in the rest of the UK.

 Overall, the paper is descriptive in nature but discusses its limitations carefully and has the advantage of laying the foundations for future research that can address more deeply the effect of confounders. To this end, the paper is very well-written paper offering a rare, up-to-date and robust view of the status if religiosity in the UK – albeit slightly skewed towards White, Christian populations. There is a good literature on the health outcomes of religiosity in the USA, especially in relation to mental health and it is commendable that the authors have addressed these issues in the UK context – although focusing on a particular region of England.

 Some key factors to consider further are the potential limitation of focusing on religious attendance as an indicator of belief or religiosity. This is because the literature on the sociology of religion has documented well how religious attendance is primarily a feature of Christian practice, for example in Islam: regular attendance at the mosque is not a compulsory facet of the faith.

 In addition, regarding the positive correlation between high socio-economic status (like degree level education) and RSBB outcome measures, there is some key literature such as by Norris and Inglehart from the 1990s about welfare security and religiosity that this paper does not refer to. Discussing the findings of this paper in relation to studies such as these, even though they are now old is still important for building the scholarly basis of this paper. Two final observations: it is not entirely clear why there is a focus on pregnant women, some more explanation of this would be helpful. The paper also does not disaggregate religious and ethnic minority groups clearly, and uses the term “non-white”. This hides the cultural nuances the study seeks to address.  The relative significance of religious outcomes such as attendance versus affiliation could also be better discussed in the paper. These are matters that could be helpful examined in a future paper.

Is the work clearly and accurately presented and does it cite the current literature?

Yes

If applicable, is the statistical analysis and its interpretation appropriate?

Yes

Are all the source data underlying the results available to ensure full reproducibility?

Yes

Is the study design appropriate and is the work technically sound?

Yes

Are the conclusions drawn adequately supported by the results?

Yes

Are sufficient details of methods and analysis provided to allow replication by others?

Yes

Reviewer Expertise:

Religion, social policy, middle east, social assistance, voluntary sector

I confirm that I have read this submission and believe that I have an appropriate level of expertise to confirm that it is of an acceptable scientific standard.

# Response

**Published:** 2023-08-18

**Authors:** Daniel Smith

**Affiliations:** University of Bristol, UK

*We thank the author for their positive review and their thoughtful and constructive comments. We have responded to the reviewer’s main suggestions in turn below, with the reviewer’s original comments in standard font and our responses in italics.*  

 One reason not explored in the paper [regarding the observed positive association between socioeconomic position and RSBB] is the close social and political association between the Church of England and the British middle class which might account for the findings regarding higher socio-economic status. This does not however, help to explain ethnic minority religious beliefs and orientations in the rest of the UK.

*Thanks for raising this important point. This has now been added to the discussion: “Alternatively, the close social, political and historical connections between the Church of England (the main religious affiliation in this population) and the British ruling and middle classes may also potentially account for the observed positive associations between socioeconomic position and RSBB.”*  

 Some key factors to consider further are the potential limitation of focusing on religious attendance as an indicator of belief or religiosity. This is because the literature on the sociology of religion has documented well how religious attendance is primarily a feature of Christian practice, for example in Islam: regular attendance at the mosque is not a compulsory facet of the faith.

*A section on this has now been added to the discussion in the paragraph on generalisability: “[…] aspects of religious practice are likely to vary by religious faith, which could not be explored in the present study, but ought to be taken into consideration in future work comparing across religions; regular religious attendance may be a more important feature of Christian practice compared to Islam, for example.”*  

 In addition, regarding the positive correlation between high socio-economic status (like degree level education) and RSBB outcome measures, there is some key literature such as by Norris and Inglehart from the 1990s about welfare security and religiosity that this paper does not refer to. Discussing the findings of this paper in relation to studies such as these, even though they are now old is still important for building the scholarly basis of this paper.

*A reference to ‘Sacred and Secular’ by Norris and Inglehart has now been added to both the introduction and the discussion.*  

 it is not entirely clear why there is a focus on pregnant women, some more explanation of this would be helpful.

*An explanation of this, and a warning that it may potentially limit generalisability to the wider adult population, has been added to the discussion: “given that participants were the parental generation of a birth cohort the focus of this study is on pregnant women and their partners. Although religiosity in this cohort is somewhat stable over time (meaning these results are unlikely to be specific to pregnancy), it is possible that different sociodemographic patterning with RSBB may be found in a more general adult population.”*  

 The paper also does not disaggregate religious and ethnic minority groups clearly, and uses the term “non-white”. This hides the cultural nuances the study seeks to address.

*Yes, unfortunately due to small sample sizes of minority ethnic groups (i.e., other than White), or religious faiths other than Christian, it was not possible to explore these important ethnic and religious nuances in any more detail. A section on this has been added to the generalisability paragraph of the discussion: “[…] due to small sample sizes we unfortunately had to combine all ‘other than White’ ethnic minority groups together, as well as all non-Christian religious affiliations (both ≤5% of the total sample). We were therefore unable to explore associations by different ethnicities, or by different religious faiths, in detail, potentially obscuring important differences between these groups”.* *Also, because it is a somewhat more inclusive term, throughout the paper we refer to ‘other than White’ ethnicities, rather than ‘non-White’ (other than in the short variable labels – e.g., ‘nonWhiteEthnic’ – in the tables and figures, which are purely for reasons of brevity, as ‘otherThanWhiteEthnic’ would have been too long).*  

 The relative significance of religious outcomes such as attendance versus affiliation could also be better discussed in the paper.

*We have now included a section in the discussion mentioning how these different religious beliefs and behaviours may have different associations with subsequent health and behavioural outcomes: “For example, much work has suggested that religious attendance, rather than other aspects of RSBB, often has stronger associations with a range of health and behavioural outcomes, including mental health, prosocial behaviour and all-cause mortality, perhaps due to enhanced social support from religious networks and the transmission of religious norms.”*

# Reviewer report: Reviewer response for version 2

**DOI:** 10.21956/wellcomeopenres.20786.r53897

**Published:** 2023-01-12

**Authors:** Tery Setiawan

**Affiliations:** 1 Faculty of Psychology, Universitas Kristen Maranatha, Bandung, Indonesia; 2 Department of Anthropology and Development Studies, Radboud University, Nijmegen, The Netherlands

I believe that the article has improved and is an important contribution to the extant literature on the relevance of religiosity in mental health. In addition, the statistical assumptions in the article have been made clear and, thus, the article should provide grounds for future works focusing on the causal relations between religiosity/spirituality dimensions and mental health.  

 I have only one minor thing for the authors to address at this point:

1. "This body of work indicates that there is no simple association between socioeconomic position and RSBB. and Tthat ..." Punctuation and spelling can be easily revised.

Is the work clearly and accurately presented and does it cite the current literature?

Partly

If applicable, is the statistical analysis and its interpretation appropriate?

Yes

Are all the source data underlying the results available to ensure full reproducibility?

Yes

Is the study design appropriate and is the work technically sound?

Yes

Are the conclusions drawn adequately supported by the results?

Partly

Are sufficient details of methods and analysis provided to allow replication by others?

Yes

Reviewer Expertise:

Social psychology, interreligious conflict, wellbeing, social identity

I confirm that I have read this submission and believe that I have an appropriate level of expertise to confirm that it is of an acceptable scientific standard.

# Response

**Published:** 2023-08-18

**Authors:** Daniel Smith

**Affiliations:** University of Bristol, UK

We are delighted with the positive review and thank the reviewer for their input. We also apologise for the length of time taken to respond to the reviewer’s comments; it took a while to find a further reviewer for this paper, and we thank this reviewer for their patience. We have now updated the minor typo/error noted by the reviewer.

# Reviewer report: Reviewer response for version 1

**DOI:** 10.21956/wellcomeopenres.19830.r53033

**Published:** 2022-12-05

**Authors:** Tery Setiawan

**Affiliations:** 1 Faculty of Psychology, Universitas Kristen Maranatha, Bandung, Indonesia; 2 Department of Anthropology and Development Studies, Radboud University, Nijmegen, The Netherlands

The study aims to explore whether demographic and socioeconomic factors are related with RSBB in the parental generation of a prospective birth cohort data (the Avon Longitudinal Study of Parents and Children; ALSPAC). Although the authors employed a great number of variables, they claimed this work to be primarily descriptive. In addition, by examining variables that may cause RSBB the authors aim to inform future scholars the plausible confounders of the topic under study. Finally, the authors also explicitly stress that although the selection of their measures lean on some theoretical frameworks, they argue that socioeconomic factors employed as predictors can also be caused by RSBB. Thus, paving the way for a causality discussion in this matter. 

 There are several points which I would like to discuss regarding the manuscript.

1. Identifying confounders or mediators or suppressors need strong theoretical background. By only relying on statistical findings, researchers may not be able to provide a sound theoretical explanation to the findings. Thus, although the study is claimed to be descriptive in nature, there should be some theoretical notions behind the selection of the variables employed in the study. 
  Further, the discussion on RSBB being the predictor or the dependent variable should involve a more in-depth literature review. After all, testing a causality in reality needs a strong theoretical background in determining the position of each variable involved. And I suppose it will be difficult to pick whether RSBB is a predictor or a dependent considering that each individual might be exposed to certain religious beliefs and behaviours during the first years of their life, while at the same time socioeconomic profile is also embedded within those first years of parenting.
2. There are many definitions of religiosity. The term should be defined to provide clear boundaries to readers.
3. There should be more justification provided on why such RSBB outcomes were the ones included in the study. Even when it is mainly because they were the only ones available in the dataset. 
4. In the Discussion section (p.11 of the pdf), "These patterns were similar, but more nuanced, for education, with higher educational attainment linearly associated with religious attendance, but non-linearly associated with religious belief and affiliation..." 
  This pattern is nowadays often observed in self-claimed religious countries, e.g., Indonesia. Can the authors provide an alternative explanation as to why this is also observed in the research area? Is religiosity becoming more vital in public sphere?
5. Still in the Discussion section (p.11), what do the authors mean by society-level differences in religiosity? Is there a different level of religiosity meaning in different layers of society? 
6. The authors are encouraged to provide more plausible theoretical explanations than merely statistical figures. I understand that a significant finding accompanied with low beta coefficient, for example, may not offer a lot to talk about. However, when discussing certain findings, the authors can further explain the different facets of religious beliefs that are available in the literature. For instance, beliefs in particularistic teachings and beliefs in religious ingroup superiority. And how these might be necessary to further understand the differences found in the relations. 

Is the work clearly and accurately presented and does it cite the current literature?

Partly

If applicable, is the statistical analysis and its interpretation appropriate?

Yes

Are all the source data underlying the results available to ensure full reproducibility?

Yes

Is the study design appropriate and is the work technically sound?

Yes

Are the conclusions drawn adequately supported by the results?

Partly

Are sufficient details of methods and analysis provided to allow replication by others?

Yes

Reviewer Expertise:

Social psychology, interreligious conflict, wellbeing, social identity

I confirm that I have read this submission and believe that I have an appropriate level of expertise to confirm that it is of an acceptable scientific standard, however I have significant reservations, as outlined above.

# Response

**Published:** 2022-12-16

**Authors:** Daniel Smith

**Affiliations:** University of Bristol, UK

*We thank the author for their review and their constructive comments. We have responded to the reviewer’s comments in turn below, with the reviewer’s original comments in standard font and our responses in italics.*   There are several points which I would like to discuss regarding the manuscript.

1. Identifying confounders or mediators or suppressors need strong theoretical background. By only relying on statistical findings, researchers may not be able to provide a sound theoretical explanation to the findings. Thus, although the study is claimed to be descriptive in nature, there should be some theoretical notions behind the selection of the variables employed in the study.
*The theoretical basis detailing why sociodemographic factors may be expected to cause RSBB is given in the third paragraph of the introduction. However, we acknowledge that this theoretical justification was not especially detailed, and have expanded upon this in the revised version: “Research has identified three broad categories of variables which may cause RSBB (see 9,10): socioeconomic, cognition/psychology and cultural transmission. In this paper we focus on the first factor (in addition to demographic factors). A socioeconomic perspective suggests that RSBB may be heightened in times of stress, uncertainty or insecurity as a way for people to explain and understand these events and find meaning in the world ^11-13^. This perspective posits that as material security increases, such as via well-functioning secular institutions, religiosity – broadly defined as encompassing a range of religious/spiritual beliefs and behaviours including religious belief, affiliation, attendance and prayer [Saraglou, 2011] – will decline. Therefore, socioeconomic and demographic factors – such as deprivation, lower social class, lower income and marginalised minority groups – are expected to be associated with heightened religiosity. Some evidence supports this view, finding a country-level association between increased material security and lower levels of religiosity ^14^, and that marginalised groups in US society, such as women, racial minorities and those from a lower socioeconomic position (SEP), are more likely to be religious ^11^. Indeed, there is evidence that lower SEP, often proxied by educational attainment, is associated with higher levels of religiosity ^15-17^. However, this effect is not replicated in all studies ^9,10,18^, and the association between socioeconomic factors and RSBB appears to vary by numerous factors, including, for example: i) by country, with a negative association between education and religiosity found overall, but the association was highly variable within countries, and in some countries was positive ^16^; ii) by religious denomination, with associations between education and religious belief variable depending on the Christian denomination ^18^ (see also ^19^); iii) by the RSBB outcome used, as in the US education often has a positive association with religious attendance, but a negative association with religious belief and frequency of prayer behaviours [Schwadel, 2011; Glaeser & Sacerdote, 2008]; and iv) by the socioeconomic factor explored, with education having a positive association with religious attendance but no association for income in the US [Glaeser & Sacerdote, 2008]. This body of work indicates that there is no simple association between socioeconomic position and RSBB. Studies may therefore need to examine this relationship on a case-by-case basis to explore the strength and direction of these associations and to understand the reasons for these divergences.”* *Furthermore, even if there is a strong theoretical justification, often these associations are not known with certainty and may differ for different studies (see the references cited in the introduction and discussion for numerous examples of this, e.g., different associations by country, or for different RSBB measures). For instance, in the present study there is a strong theoretical justification for thinking that socioeconomic factors may predict lower rates of religiosity, but here we found the opposite! We also note that theoretical justification isn’t always necessary to identify causality (see VanderWeele, 2021), although theoretical justification for a given causal effect is of course important and provides additional support for a given causal effect.*  
  Further, the discussion on RSBB being the predictor or the dependent variable should involve a more in-depth literature review. After all, testing a causality in reality needs a strong theoretical background in determining the position of each variable involved. And I suppose it will be difficult to pick whether RSBB is a predictor or a dependent considering that each individual might be exposed to certain religious beliefs and behaviours during the first years of their life, while at the same time socioeconomic profile is also embedded within those first years of parenting.
*The aim of this section was not to claim definitively that causality may be bidirectional, but rather that this is an assumption on which our study rests and should be explored in future work. For instance, we have provided some potential examples of this – e.g., RSBB potentially causing marital status and education – and justification for why this effect may be plausible, and how this can be tested and explored in future work (i.e., making use of longitudinal data; VanderWeele, 2016). However, we do not feel that a detailed literature review of whether each sociodemographic factor may plausibly cause RSBB is necessary for making this point, especially given that the discussion is already quite long.*  
2. There are many definitions of religiosity. The term should be defined to provide clear boundaries to readers.
*We have now added a broad definition of ‘religiosity’ in the introduction: “religiosity – broadly defined as encompassing a range of religious/spiritual beliefs and behaviours including religious belief, affiliation, attendance and prayer…”, and cited Saroglou (2011) to justify this definition.*  
3. There should be more justification provided on why such RSBB outcomes were the ones included in the study. Even when it is mainly because they were the only ones available in the dataset.
*We have provided additional justification for these RSBB outcomes in the methods section: “These RSBB outcomes were chosen because they cover a range of theoretically-important elements of religiosity (belief, affiliation and behaviour) and have been used extensively in previous research, allowing comparisons to previous literature.”, and cited the following references in support of this (Saroglou, 2011; McAndrew & Voas, 2011).*  
4. In the Discussion section (p.11 of the pdf), "These patterns were similar, but more nuanced, for education, with higher educational attainment linearly associated with religious attendance, but non-linearly associated with religious belief and affiliation..."
  This pattern is nowadays often observed in self-claimed religious countries, e.g., Indonesia. Can the authors provide an alternative explanation as to why this is also observed in the research area? Is religiosity becoming more vital in public sphere?
*Given the comments of both reviewers, we have provided a more extensive theoretical discussion of these results and potential explanations for these patterns of results. This new paragraph reads: “Additional research is required to understand these socially patterned results in greater detail and, in particular, why they differ from previous research and theoretical expectations, but we make some tentative suggestions here. One potential reason is that most of the previous work in this area has been conducted in the US, yet these associations are known to differ cross-culturally [Schwadel, 2015; Ruiter & van Tubergen, 2009]. The finding that education is positively associated with religious attendance is perhaps less surprising as it has been reported in the US previously [McFarland et al., 2011; Schwadel, 2011; Glaeser & Sacerdote, 2008]  – although cross-cultural analyses do suggest an overall negative association between education and religious attendance [Ruiter & van Tubergen, 2009]  – and could be explained by the known positive association between educational attainment and all forms of social interaction [Glaeser & Sacerdote, 2008]; as education is a proxy for socioeconomic position, this may also explain the positive association between religious attendance and other socioeconomic factors, such as income, occupational social class and area-level affluence. The positive associations between these socioeconomic factors and religious belief and affiliation are more surprising, however, as most previous research has found either negative [Schnabel, 2020; Hungerman, 2015] or null [Gervais et al., 2021; Willard & Cingl, 2017; Schwadel, 2011] associations with these RSBB measures. Although difficult to identify the precise reason(s), one potential explanation is the higher degree of state social support for the less affluent members of UK society [Alesina et al., 2004]. This may mean that the need for religion as a key source of emotional, social and psychological support when in a situation of material insecurity – as reported in various US samples [Schnabel, 2020; Taylor et al., 2017] – is weaker in the UK. A further, and not mutually exclusive, explanation could be due to differences in religiosity between the US and the UK, with the UK being much less religious than the US [Voas & Chaves, 2016]. Indeed, previous cross-cultural work has suggested that the negative association between education and religiosity is weaker in less religious countries, and in some cases even positive [Schwadel, 2015]; given the higher level of religious attendance among educated individuals within these societies, perhaps this attendance directly affects religious belief and affiliation [Schwadel, 2015]. These are of course very preliminary explanations, and we hope that future work will explore and understand these associations in greater detail.”.*  
5. Still in the Discussion section (p.11), what do the authors mean by society-level differences in religiosity? Is there a different level of religiosity meaning in different layers of society?
*We hope that the response above, which provides additional detail for how society-level differences – i.e., between the US (where most of the previous work was undertaken) and the UK (where the current research was conducted) – may perhaps explain the observed results.*  
6. The authors are encouraged to provide more plausible theoretical explanations than merely statistical figures. I understand that a significant finding accompanied with low beta coefficient, for example, may not offer a lot to talk about. However, when discussing certain findings, the authors can further explain the different facets of religious beliefs that are available in the literature. For instance, beliefs in particularistic teachings and beliefs in religious ingroup superiority. And how these might be necessary to further understand the differences found in the relations.
*As above, we hope that our more detailed discussion has provided sufficient detail regarding the theoretical interpretation and implications of these results.* *Literature cited in response to reviewer:* *Saroglou, V. (2011). Believing, bonding, behaving, and belonging: The big four religious dimensions and cultural variation. Journal of Cross-Cultural Psychology, 42(8), 1320-1340. * McAndrew, S., & Voas, D. (2011). Measuring Religiosity Using Surveys: Survey Question Bank Topic Overview 4. (Survey Resources Network Question Bank). University of Surrey *VanderWeele, T. J., Jackson, J. W., & Li, S. (2016). Causal inference and longitudinal data: a case study of religion and mental health. Social psychiatry and psychiatric epidemiology, 51(11), 1457-1466.* *VanderWeele, T. J. (2021). Can sophisticated study designs with regression analyses of observational data provide causal inferences?. JAMA psychiatry, 78(3), 244-246.*

# Reviewer report: Reviewer response for version 1

**DOI:** 10.21956/wellcomeopenres.19830.r51326

**Published:** 2022-08-25

**Authors:** Leor Zmigrod

**Affiliations:** 1 Department of Psychology, University of Cambridge, Cambridge, UK

This paper sought to characterize the relationships between socioeconomic indicators and religious and spiritual beliefs in a large adult sample. The sample size is commendable and the project is important and interesting. For scholars who are not familiar with ALSPAC, the title of the paper is a little inaccessible and misleading - it is easy to think that this paper concerns a longitudinal dimension which it doesn’t. The most important area that needs improvement in the current version of the paper is its theoretical basis which is unclear, and the empirical implications or applications which are not discussed. Below I offer some additional suggestions for clarifications and ways to strengthen the paper and better contextualize it for interdisciplinary scholars. This is important in order to evaluate whether the paper is making a genuine contribution to the literature.

 Firstly, although the authors caution against causal language, there are some instances when causal language is used. For instance, instead of “predictors” or “causes” (in the title and abstract and throughout the paper), “correlates” would be a more appropriate term here.

 Secondly, the rationale behind the paper is a little unclear. At the moment it seems a little like this demographic analysis was done as part of a bigger project or to set up future papers, but it’s not clear why the authors chose to look at these associations here. Having the data to look at the associations is surely useful, but what is the theoretical basis for examining these relationships? Moreover, in the third paragraph of the introduction the authors allude to the fact that the effect may be more complex but in the end of reading the paper, it wasn’t clear that nuanced relationships and sub profiles were genuinely elucidated. The poor theoretical basis of the paper is its weakest aspect and even in the conclusion there is no theoretical synthesis to help psychologists understand why these relationships exist here and why they matter or are worthy of further investigation.

 Thirdly, I would encourage the authors to add Bayes Factors to all of the core analyses. Bayes Factors can be easily computed in R using the package BayesFactor or on JASP which is free to download and user-friendly. Bayes Factors allow quantification of the evidential strength in the data for a particular hypothesis (e.g. that SES is negatively related to religious beliefs). This would substantially strengthen the paper and help add robustness to the correlational work and the limited R2 explained.

 Fourthly, the emphasis on ‘p-values’ is very odd and I’ve never seen a figure like Figure 6 that is one purely of p values with no effect sizes. An explanation (and maybe reexamination) of this approach would be valuable.

 Finally, it would be useful if the abstract offered more substantive information about the direction of the finding and what this might mean or why the discrepancies exist between the present sample and past samples. Why is education and income positively related to RSBB outcomes here but not in other samples? Are there features of the sample that explain this? Such an elucidation would help the paper be better situated in the literature.

Is the work clearly and accurately presented and does it cite the current literature?

Yes

If applicable, is the statistical analysis and its interpretation appropriate?

Yes

Are all the source data underlying the results available to ensure full reproducibility?

Partly

Is the study design appropriate and is the work technically sound?

Yes

Are the conclusions drawn adequately supported by the results?

Yes

Are sufficient details of methods and analysis provided to allow replication by others?

Yes

Reviewer Expertise:

Psychology of religion, psychology of ideology

I confirm that I have read this submission and believe that I have an appropriate level of expertise to confirm that it is of an acceptable scientific standard, however I have significant reservations, as outlined above.

# Response

**Published:** 2022-12-16

**Authors:** Daniel Smith

**Affiliations:** University of Bristol, UK

*We thank the author for their review and their constructive comments. We also apologise for the length of time taken to respond to the reviewer’s comments; it took a while to find a second reviewer for this paper, and we thank this reviewer for their patience. We have responded to the reviewer’s comments in turn below, with the reviewer’s original comments in standard font and our responses in italics.*  

 For scholars who are not familiar with ALSPAC, the title of the paper is a little inaccessible and misleading - it is easy to think that this paper concerns a longitudinal dimension which it doesn’t.

*ALSPAC is a ‘prospective cohort study’, and best practice guidelines (e.g., STROBE; https://www.strobe-statement.org/checklists/) recommend that studies “Indicate the study’s design with a commonly used term in the title or the abstract”. While we see how ‘prospective cohort study’ could indicate a potential longitudinal dimension, upon reading the paper we hope that readers will quickly realise that we do not explore these associations longitudinally.*  

 Firstly, although the authors caution against causal language, there are some instances when causal language is used. For instance, instead of “predictors” or “causes” (in the title and abstract and throughout the paper), “correlates” would be a more appropriate term here.

*There are indeed some instances in this paper where causal language is used, such as in the abstract where we say: “We explored associations between possible demographic and socioeconomic causes of religious/spiritual beliefs and behaviours”. We believe that this language is justified in this instance, as the aim of this paper is to describe various sociodemographic variables which may be used as confounders in future work; for a variable to be a confounder, it has to cause the exposure and the outcome (Hernán & Robins, 2020). If a variable is merely correlated with RSBB, it may be a confounder (if it causes RSBB), but could also be a mediator or a collider (if it is caused by RSBB), or have no causal association if the correlation is due to an unmeasured third variable; this distinction is crucial when considering causality, as adjustment for confounders removes bias while adjustment for mediators and colliders results in increased bias (VanderWeele et al., 2021).* *Given this, and the theoretical expectation that socioeconomic factors are likely to cause RSBB (e.g., references 11-13 cited in the text), we believe that the use of causal language when selecting sociodemographic confounders is warranted. Of course, there is the possibility that these sociodemographic factors may also be caused by religion (in which case they may not only be confounders, but also potential mediators or colliders), which we discuss in detail in the discussion. We hope this provides sufficient justification for our choice of causal language when discussing the choice of potential sociodemographic variables, even though – as the reviewer acknowledges – we caution against interpreting the results of the analyses presented in this paper as unbiased causal effect estimates.* *To try and make this clearer and avoid any future misunderstandings, we have altered the last sentence of the introduction from: “Establishing causal links between these variables and RSBB is beyond the scope of this paper, and our aim here is more modest: to describe these broad associations and act as a platform to inform future research in this area.” To: “While the selection of sociodemographic factors is based on causal considerations, our aim is not to estimate the unbiased causal effects between these sociodemographic variables and RSBB, and instead our aim here is more modest: to describe these broad associations and act as a platform to inform future research in this area.”.* *Regarding the term ‘predictors’ (e.g., in the title), we would contend that ‘prediction’ is a non-causal term and is largely synonymous with ‘associated’ or ‘correlated’ (see, e.g., Hernán, 2018 and Shmueli, 2010). For instance, carrying a lighter predicts – is correlated with – whether someone smokes, but it does not cause smoking behaviour.*  

 Secondly, the rationale behind the paper is a little unclear. At the moment it seems a little like this demographic analysis was done as part of a bigger project or to set up future papers, but it’s not clear why the authors chose to look at these associations here. Having the data to look at the associations is surely useful, but what is the theoretical basis for examining these relationships? Moreover, in the third paragraph of the introduction the authors allude to the fact that the effect may be more complex but in the end of reading the paper, it wasn’t clear that nuanced relationships and sub profiles were genuinely elucidated. The poor theoretical basis of the paper is its weakest aspect and even in the conclusion there is no theoretical synthesis to help psychologists understand why these relationships exist here and why they matter or are worthy of further investigation.

*The theoretical basis detailing why sociodemographic factors may be expected to cause RSBB is given in the third paragraph of the introduction. However, we acknowledge that this theoretical justification was not especially detailed, and have expanded upon this in the revised version:*

*“Research has identified three broad categories of variables which may cause RSBB (see 9,10): socioeconomic, cognition/psychology and cultural transmission. In this paper we focus on the first factor (in addition to demographic factors). A socioeconomic perspective suggests that RSBB may be heightened in times of stress, uncertainty or insecurity as a way for people to explain and understand these events and find meaning in the world ^11-13^. This perspective posits that as material security increases, such as via well-functioning secular institutions, religiosity – broadly defined as encompassing a range of religious/spiritual beliefs and behaviours including religious belief, affiliation, attendance and prayer [Saraglou, 2011] – will decline. Therefore, socioeconomic and demographic factors – such as deprivation, lower social class, lower income and marginalised minority groups – are expected to be associated with heightened religiosity. Some evidence supports this view, finding a country-level association between increased material security and lower levels of religiosity ^14^, and that marginalised groups in US society, such as women, racial minorities and those from a lower socioeconomic position (SEP), are more likely to be religious ^11^. Indeed, there is evidence that lower SEP, often proxied by educational attainment, is associated with higher levels of religiosity ^15-17^. However, this effect is not replicated in all studies ^9,10,18^, and the association between socioeconomic factors and RSBB appears to vary by numerous factors, including, for example: i) by country, with a negative association between education and religiosity found overall, but the association was highly variable within countries, and in some countries was positive ^16^; ii) by religious denomination, with associations between education and religious belief variable depending on the Christian denomination ^18^ (see also ^19^); iii) by the RSBB outcome used, as in the US education often has a positive association with religious attendance, but a negative association with religious belief and frequency of prayer behaviours [Schwadel, 2011; Glaeser & Sacerdote, 2008]; and iv) by the socioeconomic factor explored, with education having a positive association with religious attendance but no association for income in the US [Glaeser & Sacerdote, 2008]. This body of work indicates that there is no simple association between socioeconomic position and RSBB. Studies may therefore need to examine this relationship on a case-by-case basis to explore the strength and direction of these associations and to understand the reasons for these divergences.”*

*We have also added additional justification regarding the rationale of the study in the last paragraph of the introduction:*

*“This work is therefore primarily descriptive; by examining variables which may cause RSBB we can help inform the choice of confounders in future studies using these ALSPAC data. By analysing a range of RSBB outcomes and sociodemographic variables, this research will also provide a detailed exploration of how different measures of RSBB – including religious belief, affiliation and attendance – associate with various socioeconomic measures – including education, income, area-level deprivation and occupational social class. This research can assess whether these associations differ from one another, and also whether these results in a cohort of UK parents vary from those of previous research, most of which has been conducted in the US.”.* *While this is a matter of opinion, we would disagree that “it wasn’t clear that nuanced relationships and sub profiles were genuinely elucidated”. No individual study can explore every nuance, but by examining three RSBB outcomes – religious belief, religious affiliation and religious attendance – in addition to a large range of sociodemographic factors, this study provides a detailed description of these associations and some of the nuances and complexities involved. This includes differences between the various RSBB measures, as well as between the different sociodemographic exposures; for instance, in general there was a positive association between most measures of socioeconomic position and the three RSBB outcomes, but this differed for education, where associations were non-linear for religious belief and affiliation (with degree-level education associated with less religious belief and Christian affiliation), yet broadly linear and positive for religious attendance (figure 5).* *Regarding theoretical synthesis in the discussion, as this paper is primarily descriptive, we intentionally avoided making strong theoretical/causal claims as more in-depth future work is needed for this. However, we did touch upon some theoretical implications in the discussion, which we have expanded upon in the revised manuscript: “Additional research is required to understand these socially patterned results in greater detail and, in particular, why they differ from previous research and theoretical expectations, but we make some tentative suggestions here. One potential reason is that most of the previous work in this area has been conducted in the US, yet these associations are known to differ cross-culturally [Schwadel, 2015; Ruiter & van Tubergen, 2009]. The finding that education is positively associated with religious attendance is perhaps less surprising as it has been reported in the US previously [McFarland et al., 2011; Schwadel, 2011; Glaeser & Sacerdote, 2008]  – although cross-cultural analyses do suggest an overall negative association between education and religious attendance [Ruiter & van Tubergen, 2009]  – and could be explained by the known positive association between educational attainment and all forms of social interaction [Glaeser & Sacerdote, 2008]; as education is a proxy for socioeconomic position, this may also explain the positive association between religious attendance and other socioeconomic factors, such as income, occupational social class and area-level affluence. The positive associations between these socioeconomic factors and religious belief and affiliation are more surprising, however, as most previous research has found either negative [Schnabel, 2020; Hungerman, 2015] or null [Gervais et al., 2021; Willard & Cingl, 2017; Schwadel, 2011] associations with these RSBB measures. Although difficult to identify the precise reason(s), one potential explanation is the higher degree of state social support for the less affluent members of UK society [Alesina et al., 2004]. This may mean that the need for religion as a key source of emotional, social and psychological support when in a situation of material insecurity – as reported in various US samples [Schnabel, 2020; Taylor et al., 2017] – is weaker in the UK. A further, and not mutually exclusive, explanation could be due to differences in religiosity between the US and the UK, with the UK being much less religious than the US [Voas & Chaves, 2016]. Indeed, previous cross-cultural work has suggested that the negative association between education and religiosity is weaker in less religious countries, and in some cases even positive [Schwadel, 2015]; given the higher level of religious attendance among educated individuals within these societies, perhaps this attendance directly affects religious belief and affiliation [Schwadel, 2015]. These are of course very preliminary explanations, and we hope that future work will explore and understand these associations in greater detail.”.*

*We hope these revisions provide additional justification for our study, in addition to better theoretical integration with the literature. *  

 Thirdly, I would encourage the authors to add Bayes Factors to all of the core analyses. Bayes Factors can be easily computed in R using the package BayesFactor or on JASP which is free to download and user-friendly. Bayes Factors allow quantification of the evidential strength in the data for a particular hypothesis (e.g. that SES is negatively related to religious beliefs). This would substantially strengthen the paper and help add robustness to the correlational work and the limited R2 explained.

*We thank the reviewer for this suggestion, but are hesitant about changing the analyses to this extent without strong justification. For instance, although there may be some benefits to Bayes Factors, essentially p-values and Bayes Factors attempt to measure the same thing: the strength of evidence for one model/hypothesis over another (Held & Ott, 2018). This means that there is likely to be little benefit here to using Bayes Factors over p-values, especially since the p-values reported here are based on the results of likelihood ratio tests (i.e., comparing a model with vs without the sociodemographic exposure), which is conceptually analogous to how Bayes Factors would be applied. Although p-values can of course be abused and misinterpreted – e.g., by being arbitrarily dichotomised into ‘significant’ and ‘non-significant’ (which we have tried to avoid here) – given that they are more widely-used than Bayes Factors, we believe that the focus of p-values rather than Bayes Factors is appropriate for our study, and that adopting Bayes Factors would be unlikely to improve our inferences or dramatically alter our conclusions.*  

 Fourthly, the emphasis on ‘p-values’ is very odd and I’ve never seen a figure like Figure 6 that is one purely of p values with no effect sizes. An explanation (and maybe reexamination) of this approach would be valuable.

*We are somewhat puzzled by this statement, as our emphasis is not solely on p-values. While we certainly make use of p-values as a measure of evidence against the null hypothesis, we clearly provide measures of effect sizes via pseudo-R ^2^ values (figure 3 for mothers, figure S14 for partners), in addition to the relative risk ratio regression estimates from the multinomial models (with some results presented in the main text – e.g., figures 4 and 5 – and full results in the supplementary information). We made use of all of this information when interpreting these results, and clearly state this in the methods (“To provide a single p-value for each model to assess model fit, we ran two sets of likelihood ratio tests for each exposure-outcome combination: the first assessed whether inclusion of the exposure improved model fit relative to an age-only model (or an empty model, where age was the exposure); the second assessed whether inclusion of an interaction term between age and the exposure improved model fit relative to the model with no interaction. […] To give an indication of the increase in model fit resulting from inclusion of the exposure, we calculated the difference in McFadden’s pseudo-R2 value between the model with vs without the exposure (or with vs without the interaction term, for interaction models).”). We also refer to this in the results (“Thus, although the majority of exposures were below the Bonferroni-adjusted p-value threshold, the amount of variance explained by these exposures is likely to be small”) and the discussion (“Despite some highly significant associations (as indicated by p-values), the estimated variance explained using pseudo-R ^2^ values was relatively low, with a maximum value of 2.4%, highlighting that relatively little of the variation in RSBB is explained by individual demographic and socioeconomic factors.”).* *Given the number of comparisons made, we felt that a figure of all the p-values (like figure 2), and a separate figure of all the effect sizes (like figure 3), was the clearest way to present this information. We also note that this kind of p-value plot is common in other areas where the focus is on summarising a large number of comparisons, and is similar to Manhattan-style plots (* *https://en.wikipedia.org/wiki/Manhattan_plot* *) for genome-wide (Turner, 2014) or exome-wide (Hernandez-Ferrer et al., 2019) association studies.*  

 Finally, it would be useful if the abstract offered more substantive information about the direction of the finding and what this might mean or why the discrepancies exist between the present sample and past samples. Why is education and income positively related to RSBB outcomes here but not in other samples? Are there features of the sample that explain this? Such an elucidation would help the paper be better situated in the literature.

*We have altered the conclusion section of the abstract, from: “These findings demonstrate that numerous demographic and socioeconomic factors are associated with RSBB in this population. While additional work is needed to assess whether any of these associations are causal, this descriptive paper can help inform future studies using this data by considering appropriate confounders and thus attempt to minimise bias that confounding may introduce.” To: “The observed positive association between socioeconomic position and RSBB is contrary to much previous theoretical and empirical work. Potential reasons for these differences are discussed, including cross-cultural variation in religiosity and state support, and differences between RSBB measures. This descriptive paper can also help inform future studies using these data regarding the consideration of appropriate confounders.”.*  

*Literature cited in response to reviewer:* *Held, L., & Ott, M. (2018). On p-values and Bayes factors. Annual Review of Statistics and Its Application, 5(1), 593-419.* *Hernán, M. A. (2018). The C-word: scientific euphemisms do not improve causal inference from observational data. American journal of public health, 108(5), 616-619.* *Hernán MA, Robins JM (2020). Causal Inference: What If. Boca Raton: Chapman & Hall/CRC.* *Hernandez-Ferrer, C., Wellenius, G. A., Tamayo, I., Basagaña, X., Sunyer, J., Vrijheid, M., & Gonzalez, J. R. (2019). Comprehensive study of the exposome and omic data using rexposome bioconductor packages. Bioinformatics, 35(24), 5344-5345.* *Shmueli, G. (2010). To explain or to predict?. Statistical science, 25(3), 289-310.* *Turner, S. D. (2014). qqman: an R package for visualizing GWAS results using QQ and manhattan plots. Biorxiv, 005165.* *VanderWeele, T. J., Rothman, K. J., & Lash, T. L. (2021). Confounding and Confounders. Modern epidemiology, 263-286.*
